# Simultaneous observation of auroral substorm onset in Polar satellite global images and ground-based all-sky images

**DOI:** 10.1186/s40623-018-0843-3

**Published:** 2018-05-04

**Authors:** Akimasa Ieda, Kirsti Kauristie, Yukitoshi Nishimura, Yukinaga Miyashita, Harald U. Frey, Liisa Juusola, Daniel Whiter, Masahito Nosé, Matthew O. Fillingim, Farideh Honary, Neil C. Rogers, Yoshizumi Miyoshi, Tsubasa Miura, Takahiro Kawashima, Shinobu Machida

**Affiliations:** 1 Institute for Space-Earth Environmental Research, Nagoya University, Nagoya, Aichi Japan; 2 Finnish Meteorological Institute, Helsinki, Finland; 3 Department of Atmospheric and Oceanic Sciences, University of California, Los Angeles, CA USA; 4 Department of Electrical and Computer Engineering and Center for Space Physics, Boston University, Boston, MA USA; 5 Korea Astronomy and Space Science Institute, Daejeon, South Korea; 6 Space Sciences Laboratory, University of California, Berkeley, CA USA; 7 School of Physics and Astronomy, University of Southampton, Highfield, Southampton UK; 8 Data Analysis Center for Geomagnetism and Space Magnetism, Graduate School of Science, Kyoto University, Kyoto, Japan; 9 Space and Planetary Physics, Lancaster University, Bailrigg, Lancaster UK

**Keywords:** Substorm, Auroral breakup, Aurora, Substorm onset, Global images, All-sky images

## Abstract

Substorm onset has originally been defined as a longitudinally extended sudden auroral brightening (Akasofu initial brightening: AIB) followed a few minutes later by an auroral poleward expansion in ground-based all-sky images (ASIs). In contrast, such clearly marked two-stage development has not been evident in satellite-based global images (GIs). Instead, substorm onsets have been identified as localized sudden brightenings that expand immediately poleward. To resolve these differences, optical substorm onset signatures in GIs and ASIs are compared in this study for a substorm that occurred on December 7, 1999. For this substorm, the Polar satellite ultraviolet global imager was operated with a fixed-filter (170 nm) mode, enabling a higher time resolution (37 s) than usual to resolve the possible two-stage development. These data were compared with 20-s resolution green-line (557.7 nm) ASIs at Muonio in Finland. The ASIs revealed the AIB at 2124:50 UT and the subsequent poleward expansion at 2127:50 UT, whereas the GIs revealed only an onset brightening that started at 2127:49 UT. Thus, the onset in the GIs was delayed relative to the AIB and in fact agreed with the poleward expansion in the ASIs. The fact that the AIB was not evident in the GIs may be attributed to the limited spatial resolution of GIs for thin auroral arc brightenings. The implications of these results for the definition of substorm onset are discussed herein.
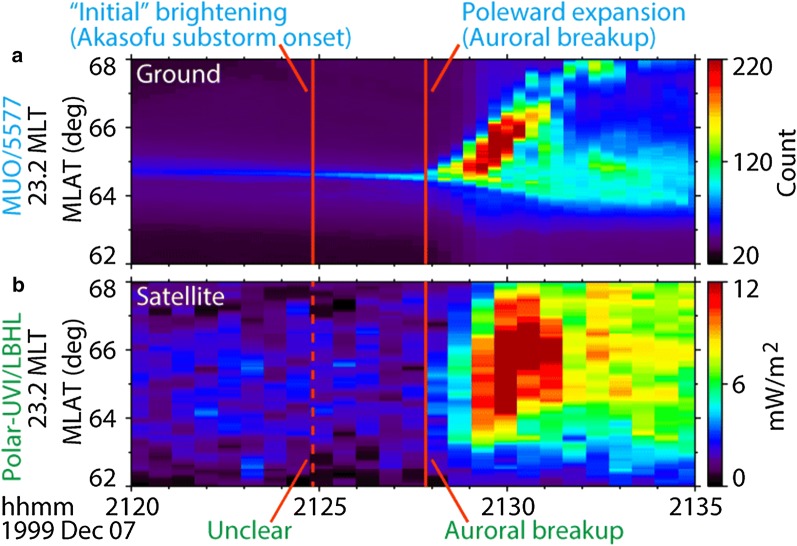

## Introduction

A substorm refers to the explosive release of stored energy in the magnetotail (e.g., Akasofu 1977). It is necessary to identify substorm onsets with an accuracy of at least a few minutes to determine the triggering mechanism of the substorm, such as magnetic reconnection in the magnetotail. Substorm onsets have often been identified with a sudden auroral brightening in both satellite-based global images (GIs) and ground-based all-sky images (ASIs). Thus, this sudden brightening should exhibit similar features in both GIs and ASIs. However, the shape of the observed sudden brightening actually differs between the two image types.

**Fig. 1:**
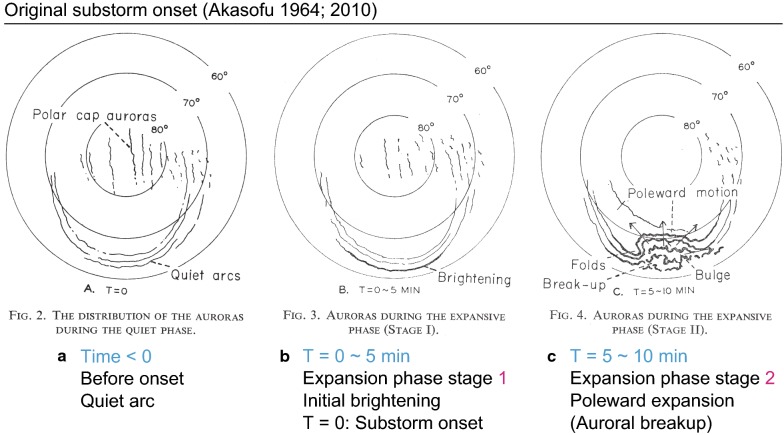
Original illustrations and figure captions of the Akasofu substorm onset (Akasofu 1964). Reprinted with permission from Elsevier. Clarifications are added on the bottom of the figure. The illustrated time sequence was proposed on the basis of 1-min resolution ground all-sky images (ASIs). Auroral emissions in the polar ionosphere above $60^\circ$ magnetic latitude are illustrated. $T = 0$ min represents the time of the Akasofu substorm onset. **a** ${T} < 0$: Quiet time. Quiet-time auroral arcs are shown. **b** *T* = 0 ~ 5 min: Akasofu initial brightening (AIB; i.e., Akasofu substorm onset), starting at $T = 0$. Also called Stage 1 of the substorm expansion phase. This brightening is wide in longitude without poleward expansion. **c** $T = 5 \sim 10$ min: Poleward expansion, starting at $T = 5$. Also called Stage 2 of the substorm expansion phase. The two-stage development has also been illustrated in later studies (e.g., Akasofu et al. 2010) and is essential in this Akasofu substorm onset

The substorm concept is a comprehensive understanding of the auroral breakup phenomenon. Auroral breakup is used to refer to a sudden and intense increase in the brightness and motion of an aurora in the polar ionosphere (e.g., Elvey 1957; Akasofu 1963). Akasofu (1964) captured auroral breakup images using widely distributed ground all-sky cameras with a time resolution of 1 min. He found breakup-associated new features and termed them collectively as a substorm. In particular, he identified the stage in which the sudden auroral brightenings are wide in longitude as initial brightening (IB; Fig. 1). It should be noted that the IB is recognized as wide when considered on a timescale of a few minutes. On much shorter timescales, the same IB may appear localized at the very beginning and expand quickly in longitude (e.g., Akasofu 2012). The auroral bead phenomenon (e.g., Liang et al. 2008) is presumably one such type of detailed feature of this wide brightening.

The IB does not merely imply the “first” observed brightening; it also describes the time of the substorm onset (e.g., Akasofu et al. 2010). We refer to this phenomenon as Akasofu IB (AIB) in the present study to avoid confusion. Accordingly, when a substorm onset is specifically identified on the basis of AIB, this type is referred to as “Akasofu substorm onset.” The AIB is followed by poleward expansion of the aurora a few minutes later in the original substorm model (Akasofu 1964). That is, Akasofu (1964) found that the auroral breakup phenomenon (e.g., Akasofu 1963) begins with a two-stage development. The term “auroral breakup” has been used in various contexts. In the present study, we define auroral breakup as an auroral brightening immediately followed by poleward expansion. In this context, auroral breakup is delayed as compared with the Akasofu substorm onset.

In contrast to Akasofu (1964), the substorm onset is not recognized as being elongated along longitudes but is instead localized in statistical studies of GIs as follows. Frey et al. (2004); Frey and Mende (2006) identified substorm onsets by “a clear local brightening of the aurora” within GIs observed by the far ultraviolet imager (FUV) onboard the Imager for Magnetopause-to-Aurora Global Exploration (IMAGE) satellite. Liou (2010) identified substorm onsets by “a sudden brightening of the aurora” within GIs observed by the ultraviolet imager (UVI) onboard the Polar satellite. Practically, Liou (2010) first identified an auroral bulge and then traced it back in time to identify its original instance and location. Thus, the sudden brightening appears to have been recognized as relatively localized and immediately followed by poleward expansion.

This localized onset in GIs may be confused as corresponding to the localized brightening observed in high time resolution ASIs of a few seconds. However, the longitudinally localized brightening in such ASIs expands quickly in the east–west direction (e.g., Liang et al. 2008) to form a longitudinally extended brighter aurora (i.e., AIB) before the poleward expansion. Because even such a longitudinally extended aurora is not mentioned in these onset identifications made with GIs, the initially less intense localized brightening in ASIs is not likely evident in GIs. In summary, substorm onsets in GIs are not likely to correspond directly to the Akasofu substorm onsets in ASIs.

Because GIs have limited sensitivities compared with ASIs, small or weak signatures are not evident in them. This widely established caveat implies that the time of the observed first brightening is expected to be delayed in GIs compared with that in ASIs. In contrast, the possible delay of GI onsets with respect to ASI onsets has been expected to be small, at less than $\sim 1\hbox { min}$ (e.g., Liou 2010). Moreover, substorm onsets in GIs are simultaneous or even earlier than Pi2 pulsations (Liou et al. 1999). Thus, the impact of the caveat on the identification of substorm onset time in GIs may not be significant.

This possible delay should be clarified by using simultaneous ASI and GI observations. The onsets often begin outside ASI field of view (e.g., Shiokawa et al. 2005; Yago et al. 2007). Three fortunate cases with onsets inside the ASI field of view have been reported (Tagirov et al. 1998; Bristow et al. 2003; Donovan et al. 2006). Tagirov et al. (1998) and Bristow et al. (2003) recognized that the onsets are simultaneous between ASIs and GIs on a timescale of 1 min. In contrast, Donovan et al. (2006) suggested that a GI onset is delayed by a few minutes. This delay is comparable to the time resolution (2 min) of the IMAGE satellite FUV images and thus is not conclusive.

Because the Polar/UVI usually changes filters (e.g., Tagirov et al. 1998; Bristow et al. 2003), detailed comparisons with ASIs within 3 min are generally difficult. However, the Polar/UVI is sometimes operated under the fixed-filter mode. This mode enabled us to compare simultaneous GIs and ASIs with a practical time resolution of less than 1 min for the first time.

The purpose of the present study is to clarify the difference in the observed substorm onset between ASIs and GIs. Compared with GIs, the regional images from the Reimei satellite provide more consistent timing information with ASIs (Frey et al. 2010; Zou et al. 2010). In the present study, “GIs” specifically refer to images with a practical spatial resolution of $\sim 50\hbox { km}$ or slightly worse, such as Polar/UVI or IMAGE/FUV images. These GIs were used to construct extensive substorm onset lists (Frey and Mende 2006; Liou 2010) that are publically available. Thus, substorm onsets identified in these GIs are practically standard references and have been further compared widely with other signatures, particularly with tail reconnection (e.g., Baker et al. 2002; Kepko et al. 2004; Miyashita et al. 2009). New results on substorm onsets with ASIs should be compared with past results with GIs to gain a comprehensive understanding. Thus, it is critical to clarify the difference between ASIs and GIs. In particular, we aim to understand the absence of the two-stage development in GIs.

Accordingly, we suggest that substorm onsets that are identified using solely GIs do not necessarily correspond to the Akasofu substorm onset in ASIs; rather, they correspond to the subsequent poleward expansion. We will also show that traditional geomagnetic bays and midlatitude Pi2 pulsations correspond to poleward expansion rather than Akasofu substorm onset. These results require an update of the interpretation of the time difference between the substorm onset and reconnection signatures reported in previous studies.

## Data set

### Polar satellite global images

The Polar satellite ultraviolet imager (UVI) (Torr et al. 1995) provides global imaging of auroras. UVI GIs are captured in the $\mathrm{N_{2}}$ Lyman–Birge–Hopfield long (LBHL, $\sim 170\hbox { nm}$) and short (LBHS, $\sim 150\hbox { nm}$) wavelengths and the OI $\sim 130.4$ and $\sim 135.6\hbox { nm}$ wavelengths. In particular, the LBHL images monitor the energy flux of precipitating keV-range electrons (e.g., Lummerzheim et al. 1997) and thus can be compared with green-line (557.7 nm) ASIs during substorms. The LBHS images are less useful for this purpose because LBHS emissions are absorbed by the atmosphere en route to the imager, and this absorption depends on the average energy of precipitating auroral electrons. The UVI captures four or five images in each 184-s cycle. The filter and the exposure period usually vary during this 3-min cycle; thus, the practical time resolution is usually 3 min for the same filter and exposure.

In the present study, the UVI captured images with a fixed wavelength (LBHL) and exposure mode at 36.8 s, which enabled a higher practical time resolution of 36.8 s than the usual 3 min. This 37-s resolution is expected to be marginally sufficient for resolving the two stages of the substorm onset sequence, which are presumably separated by a few minutes. The spatial resolution (i.e., 1 pixel) of images ($200 \times 228\hbox { pixels}$) is $\sim 37 \times 31\hbox { km}$ when viewed vertically from the Polar satellite with its apogee of $9R_{E}$. Practically, UVI images are smeared by approximately $\pm \,5$ pixels owing to satellite-spin-associated wobbling (e.g., Germany et al. 1998; Frank et al. 2001a). The emission altitude was assumed to be 120 km from the ground. Slant path brightness enhancements were corrected by using an empirical model similar to a cosine curve (e.g., Germany et al. 1998). The emission brightness was converted to the energy flux of the precipitating electrons that cause auroras by using 130 R per $\mathrm{mW}\ \mathrm{m}^{-2}$, referring to the results of Galand and Lummerzheim (2004).

**Fig. 2:**
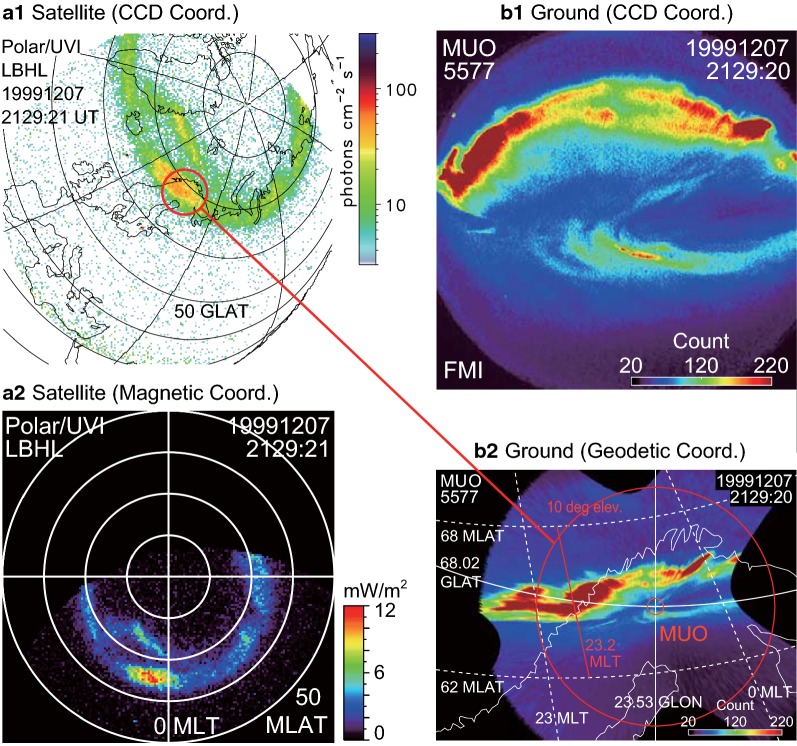
Example of simultaneous satellite-based global images and ground-based all-sky images. These images were observed at 2129 UT on December 7, 1999. **a1** Polar satellite global image in the raw (CCD) coordinates with an overlaid geographical map. Auroral emission at ultraviolet 170 nm (LBHL) is shown. **a2** The same satellite image as (**a1**) but in the magnetic coordinates (i.e., the modified APEX coordinates). **b1** Ground-based all-sky image observed at Muonio (MUO, 64.6 MLAT, 105.2 MLON, $68.02^\circ \hbox {N}$, $23.53^\circ \hbox {E}$) in Lapland, Finland, in the raw (CCD) coordinates. Auroral emission at 557.7 nm (green-line) is shown. **b2** The same ground image as (**b1**) but in the geodetic coordinates (the azimuthal equidistant projection), trimmed at the elevation angle of $5^\circ$. The dashed white lines indicate MLT and MLAT reference lines. The red line indicates the substorm onset MLT (23.2 h), from where auroral keograms were made later. The red circles in **a1** and **b2** indicate the field of view of the ground images for the elevation angle of $10^\circ$, corresponding to a diameter of $\sim 1000\hbox { km}$ ($\sim 9^\circ$ along latitudes) to the assumed emission altitude of 110 km

Figure 2a1 shows an example of the UVI image in the raw charged couple device (CCD) coordinates with an overlaid geographical map. The magnetic coordinates (Fig. 2a2) have often been used to show GIs in previous substorm studies. We calculated the magnetic latitude (MLAT) and longitude (MLON) in degrees and the magnetic local time (MLT) in hours of the modified magnetic apex coordinates (Richmond 1995) for a reference altitude of 110 km using the IGRF-12 (Thebault et al. 2015) model.

### All-sky images at Muonio (65 MLAT)

The satellite images were compared with ASIs observed at Muonio (MUO: 64.6 MLAT, 105.2 MLON, $68.02{^{\circ }}\hbox {N}$, $23.53{^{\circ }}\hbox {E}$) in Lapland, Finland (e.g., Fig. 2b1 and b2). The red circle in Fig. 2b2 and a1 with a diameter of $\sim 1000\hbox { km}$ roughly indicates the field of view of the imager. This intensified CCD all-sky camera is maintained by the Finnish Meteorological Institute (e.g., Syrjäsuo et al. 1998; Sangalli et al. 2011; Partamies et al. 2015).

We used the green-line (557.7 nm) images captured every 20 s with an exposure time of 1 s. Figure 2b1 shows an example image in raw (CCD) coordinates. The $512 \times 512$ pixel images correspond to $\sim 1\hbox { km}$ resolution overhead at an assumed emission altitude of 110 km. The geodetic coordinates (Fig. 2b2) have often been used to show ASIs in previous substorm studies.

## Observations

### Satellite-based global images

**Fig. 3:**
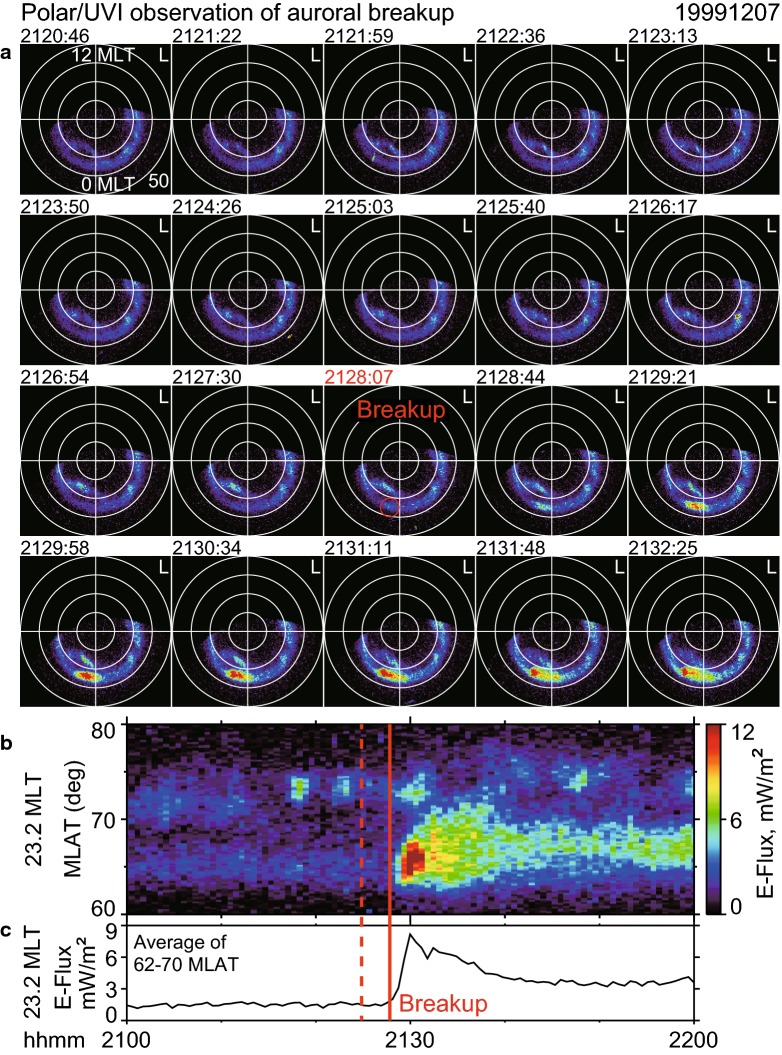
Polar satellite ultraviolet observations of an auroral breakup on December 7, 1999. Auroral brightness at a wavelength of 170 nm (LBHL) is shown after projection in the modified APEX magnetic coordinates at an altitude of 110 km in the polar ionosphere. The auroral brightness is converted to the corresponding energy flux of precipitating electrons that cause auroras. **a** Time series of full-time (36.8-s) resolution images shown in false color from left to right. The time labels of images were chosen as the center of the image accumulation period (36.8 s for the present case). An auroral breakup (red circle, 23.2 MLT, $64.6^{\circ }$ MLAT) is first seen in the panel labeled 2128:07 UT in red. Because the previous image was taken at 2127:30 UT, we estimate that the breakup began at 2127:49 UT (i.e., the center time of the two images). **b** Auroral keogram sliced along the onset meridian (23.2 MLT, ± 0.2 h average). **c** Auroral brightness averaged over 23.0–23.4 MLT and 62–70 MLAT. The solid red line in **b** and **c** indicates the breakup at 2127:49 UT. The dashed red line indicates 2124:50 UT

Figure 3a shows the time sequence of Polar/UVI images in the MLT–MLAT polar coordinates. An auroral brightening was first observed in the panel labeled in red at 2128:07 UT, which is midpoint of the 36.8-s exposure time. The brightening was located around [23.2 MLT, 64.6 MLAT] as indicated by the red circle. The time of the previous image was 2127:30 UT, just prior to the brightening event. We consider the average of these two times, 2127:49 UT, as the beginning of the auroral brightening event.

This brightening appears to be localized at the beginning of the event and was immediately followed by poleward expansion, as shown in later panels. These are typical signatures of auroral breakup, or substorm onset, in GIs (e.g., Liou et al. 2000; Frey et al. 2004). This result is also shown in the auroral keogram (Fig. 3b), where the onset at 2127:49 UT is marked by a solid vertical line. No other brightenings were evident before the onset, as also indicated by the average of the keogram data between 62 and 70 MLAT (Fig. 3c), particularly around 3 min (dashed vertical line) before the onset. Because the possible two-stage development was not identified, it is unclear solely from the GIs whether this substorm onset is the Akasofu substorm onset.

### Ground-based all-sky images

**Fig. 4:**
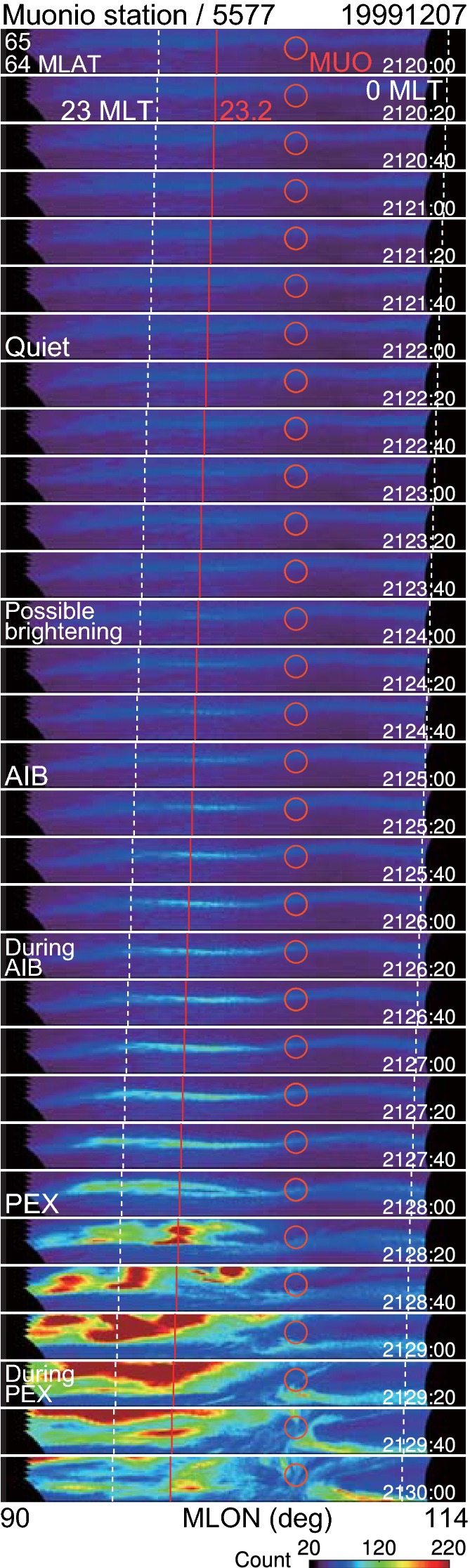
Ground-based all-sky images (ASIs) near the substorm onset location on December 7, 1999. Auroral brightness at a wavelength of 557.7 nm (green-line) observed at Muonio (MUO) in Finland is shown. Time sequence of full-time-resolution (20-s) images from top to bottom in MLON–MLAT coordinates between 64 and 65 MLAT. The red circle indicates the location of MUO. The red line indicates 23.2 MLT, the approximate location of the initiation of the Akasofu initial brightening (AIB) and the poleward expansion

**Fig. 5:**
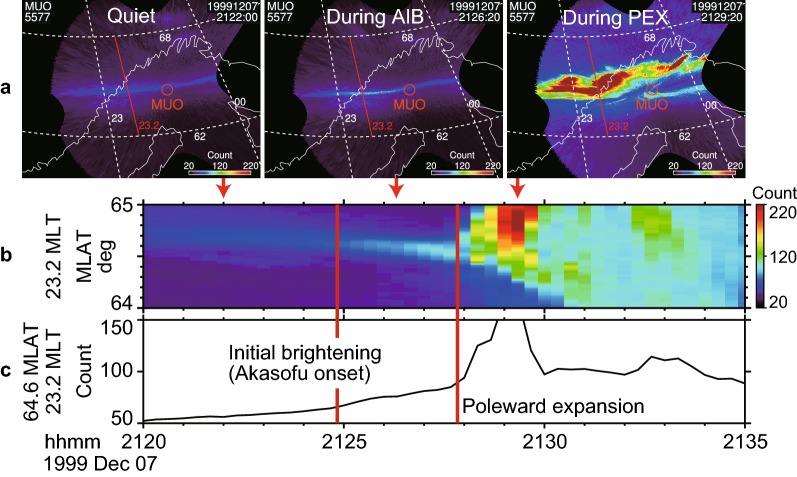
Ground-based all-sky images (ASIs) near the substorm onset location on December 7, 1999. Auroral brightness at a wavelength of 557.7 nm (green-line) observed at Muonio (MUO) in Finland is shown. **a** Selected images that represent three intervals as labeled, shown in the geodetic coordinates. The white reference lines represent MLTs and MLATs. The red line indicates 23.2 MLT, the approximate location of the initiation of the Akasofu initial brightening (AIB) and the poleward expansion. **b** Auroral keograms sliced along the onset meridian (23.2 MLT, ± 0.2 h average), which correspond to the red lines in **a**. **c** Time series of auroral brightness near the onset location (23.2 MLT, 64.6 MLAT), averaged over 23.0–23.4 MLT and 64.5–64.7 MLAT. The red vertical lines in **b** and **c** indicate the times of the AIB (2124:50 UT) and the poleward expansion (2127:50 UT) in the ASIs

In contrast, AIB was observed in the ground ASIs (Figs. 4 and 5) captured at the MUO station. Figure 4 shows the time sequence of full-time 20-s resolution images. Figure 5a shows three selected images that represent moments during the quiet time, initial brightening, and poleward expansion. A brightened auroral arc is evident in the 2126:20 UT image in Figs. 4 and 5a, but it is subjective to determine precisely when it began. Tracing this arc back in time starting at 2126:20 UT, we determined that the arc brightening began at the 2125:00 UT image in Fig. 4. This detailed selection of the start time is moderately supported by the auroral keogram (Fig. 5b) and by the auroral brightness near the onset location (Fig. 5c), which shows a small enhancement in its increase rate.

However, faint spots appear before 2125:00 UT in Fig. 4. In particular, a spot at (23.18 MLT, 64.6 MLAT) near the onset MLT in the 2124:00 UT panel may be another possible candidate for the start of the brightening. Thus, the selection of the 2125:00 UT image as the first brightening is subjective for approximately 1 min. The auroras show faint azimuthally separated structures near the onset MLT, e.g., 2124:40 UT and 2126:20 UT panels. These structures are presumably consistent with auroral beading (e.g., Donovan et al. 2006; Liang et al. 2008), although their signals are weak in this particular event.

The first brightening identified above was centered at [23.2 MLT, 64.6 MLAT] in the 2125:00 UT image and spanned approximately between 22.8 and 23.6 MLT in the 2126:00 UT image. Because this brightening occurred simultaneously within a few minutes across a wide longitude, it can be interpreted to be the AIB that was used to define the substorm onset by Akasofu (1964). It should be noted that we do not specifically require the AIB to be as wide as those illustrated in Akasofu (1964) and Akasofu et al. (2010), which span 4–6 h in MLT and would be typically too wide before the poleward expansion. Because the original images were captured at 20-s intervals, we assumed that the AIB began at 2124:50 UT, 10 s before 2125:00 UT.

The brightened arc shows a small split at 23.1 MLT in the 2127:40 UT image, but the poleward expansion has not yet started in this image and in the keogram. The poleward expansion actually begins in the next image, at 2128:00 UT, when the bright part (22.8–23.5 MLT) of the auroral arc began to split in the northern direction. The resultant poleward arc expanded further poleward, as shown in the 2129:00 UT panel. We assumed that this poleward expansion began at 2127:50 UT, 10-s before 2128:00 UT. An associated auroral brightening occurred simultaneously or in the previous image at 2127:40 UT, depending on the subjectivity. Because this second brightening was followed immediately by the poleward expansion, it is considered in the present study to be an auroral breakup.

In summary, the AIB was identified at 2124:50 UT with a subjectivity of approximately 1 min. Mende et al. (2009) also reported that AIB can be too gradual to identify within $\sim 10\hbox {-s}$ accuracy. The increasing rate of auroral brightness was approximately constant during the AIB (Fig. 5c). The poleward expansion was identified at 2127:50 UT, which is delayed from the AIB by at least 2 min and most likely 3 min. Thus, the two-stage development was evident in the ASIs.

### Comparisons of ground and satellite images

**Fig. 6:**
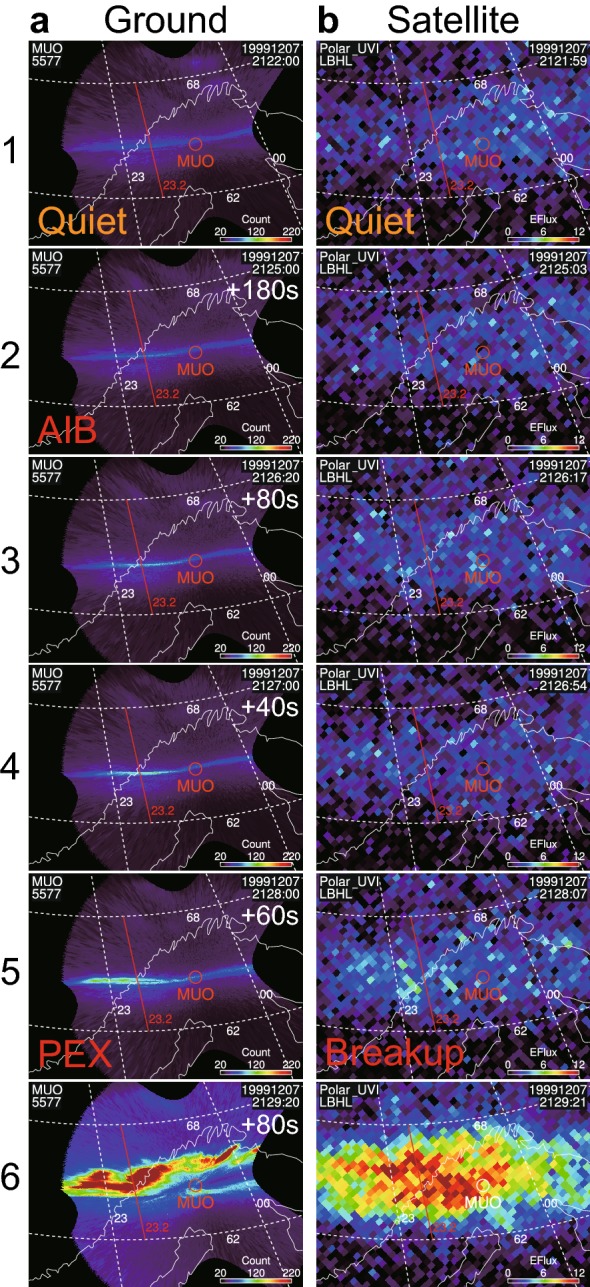
Comparison of **a** ground-based and **b** satellite-based auroral images on December 7, 1999. The time sequence of selected auroral images is shown from top to bottom. All images are projected to the same area in geodetic coordinates. **a** Ground-based all-sky images (ASIs; 557.7 nm) at the Muonio station (MUO) in Finland. These ASIs were selected to show the observed instances **a1** during the quiet interval, **a2** at the start of Akasofu initial brightening (AIB), **a3**–**a4** during AIB, **a5** at the start of poleward expansion, and **a6** during poleward expansion. **b** Global images (170 nm) taken by the Polar satellite ultraviolet imager (UVI). Each image was selected to form a pair with an ASI in **a** within 7 s. A comparison of **a** and **b** reveals that the longitudinally extended brightening (AIB) can be marginally observed in **a2** and is evident in **a3**–**a4** but not in **b2**–**b4**. In contrast, the brightening **a5** that corresponds to the beginning of the poleward expansion was simultaneously observed in **b5**

Figure 6 shows simultaneous comparisons of ground and satellite images. Figure 6a shows ground ASIs observed at MUO, projected in the geodetic coordinates. These ASIs were selected with 40–180 s separations to represent the observed instances (a1) before onset, (a2) at the start of the AIB, (a3 and a4) during the AIB, (a5) at the start of the poleward expansion, and (a6) during the poleward expansion.

Figure 6b shows the corresponding Polar UVI images for the same fixed area as that in Fig. 6a. Each image was selected to correspond to an ASI (Fig. 6a) within 7 s. A comparison of Fig. 6a and b reveals the poleward expansion in the ASIs (a5 and a6) was simultaneously observed in the GIs (b5 and b6), although the GIs appear smeared by satellite-spin-associated wobbling. In contrast, the AIB in the ASIs (a2, a3, and a4) was not evident in the corresponding GIs (b2, b3, and b4).

**Fig. 7:**
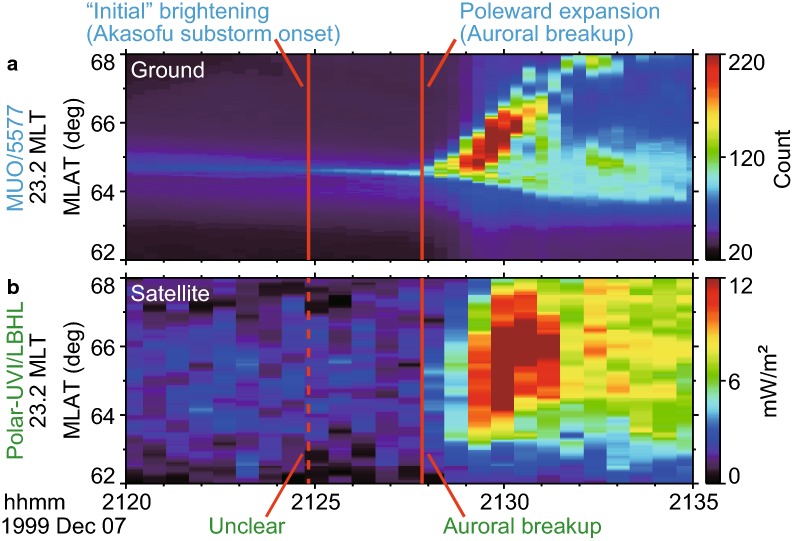
Comparison of **a** ground-based and **b** satellite-based observations of auroras on December 7, 1999. The time series of full-time-resolution images were sliced along the onset meridian (23.2 MLT, ± 0.2 h average) and are shown as auroral keograms. The red vertical lines indicate the times of the Akasofu initial brightening (AIB) and the poleward expansion, which were identified in the original ground-based images. The poleward expansion (i.e., auroral breakup) at 2127:50 UT was simultaneously observed in both ASIs and GIs. The AIB was observed at 2124:50 UT in the ASIs but was not evident in the GIs against noise-level fluctuations

These characteristics were also observed in the keograms (Fig. 7), where slices of images at 23.2 MLT between 62 and 68 MLAT are shown. Again, poleward expansion was observed at about 2127:50 UT both in the (a) ASIs and (b) GIs. In contrast, the AIB (i.e., Akasofu substorm onset), which was observed at 2124:50 UT in the ASIs, was not evident in the GIs until the poleward expansion began.

In summary, the counterpart of the AIB was not evident in the GIs. Consequently, the observed first brightening in the GIs corresponded to the second brightening in the ASIs (i.e., poleward expansion). Therefore, we suggest that the substorm onsets in the GIs and ASIs represent different stages of substorms, particularly when these onsets are identified independently (e.g., Figs. 3 and 4).

### Solar wind and geomagnetic indices

**Fig. 8:**
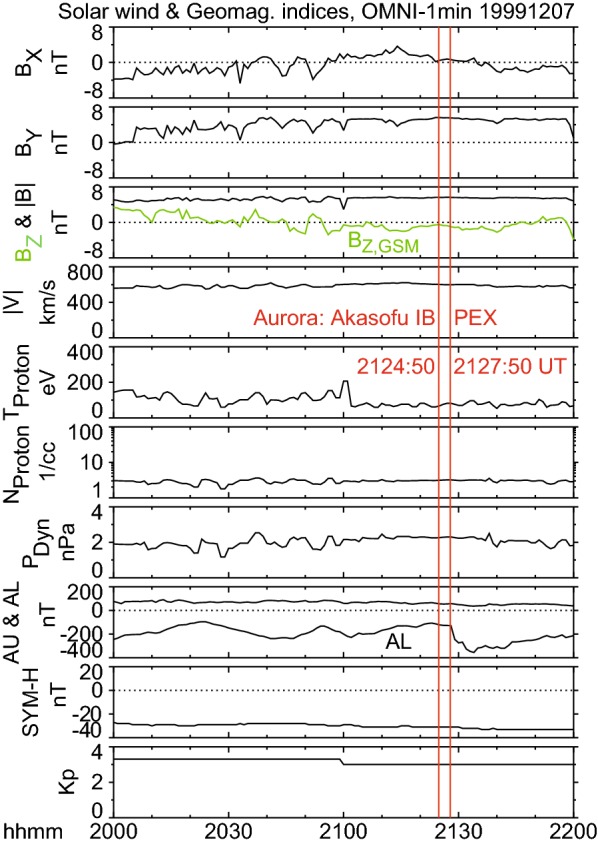
Solar wind parameters and geomagnetic indices on December 7, 1999. These data were obtained from the Operating Missions as Nodes on the Internet (OMNI) data set. The red vertical lines indicate the Akasofu initial brightening (AIB; 2124:50 UT) and the poleward expansion (2127:50 UT) identified by using all-sky images (ASIs). The solar wind parameters were time-shifted with respect to the bow shock nose. Geocentric solar magnetospheric (GSM) coordinates were used. The interplanetary magnetic field (IMF) was weakly southward ($B_{z}~\sim ~-1~\hbox {nT}$) and strongly duskward ($B_{y}~\sim ~6~\hbox {nT}$), indicating moderately favorable conditions for the occurrence of substorms. The *AL* index (i.e., westward ionospheric current) began to develop at 2129 UT, which is closer in time to the poleward expansion than to the AIB in the ASIs

Figure 8 shows the solar wind and geomagnetic indices obtained from the Operating Missions as Nodes on the Internet (OMNI) (King and Papitashvili 2005) 1-min resolution data. The north–south component of the interplanetary magnetic field (IMF) was weakly southward between 0 and − 3 nT from 2054 UT, or about 30 min prior to the AIB, to 2145 UT. The dawn–dusk component and the magnitude of IMF were relatively strong during this interval, at 6 nT duskward. The solar wind speed was relatively high at 600 km/s, although the plasma density was relatively low at $2/\mathrm{cm}^{3}$, resulting in a normal dynamic pressure at 2 nPa.

The geomagnetic condition was moderately disturbed during the 2-h period, as shown by the *Kp* index (3+ to 3) and *SYM-H* indices ($\sim {-}\,30\hbox {nT}$). This disturbed interval belonged to a co-rotation interaction region-type weak (peak $\sim {-}\,40\hbox {nT}$) magnetic storm that began four days prior at around 9 UT on December 3, 1999 (not shown). The *AL* index began to develop at 2129 UT (Fig. 8), 1 min after the poleward expansion, and 4 min after the Akasofu substorm onset in the ground ASIs (Fig. 7). The *AL* was − 127, − 128, and − 245 nT at 2127, 2128, and 2129 UT, respectively, and reached its peak value of − 355 nT at 2134 UT.

### Negative bays in the ground magnetic field

**Fig. 9:**
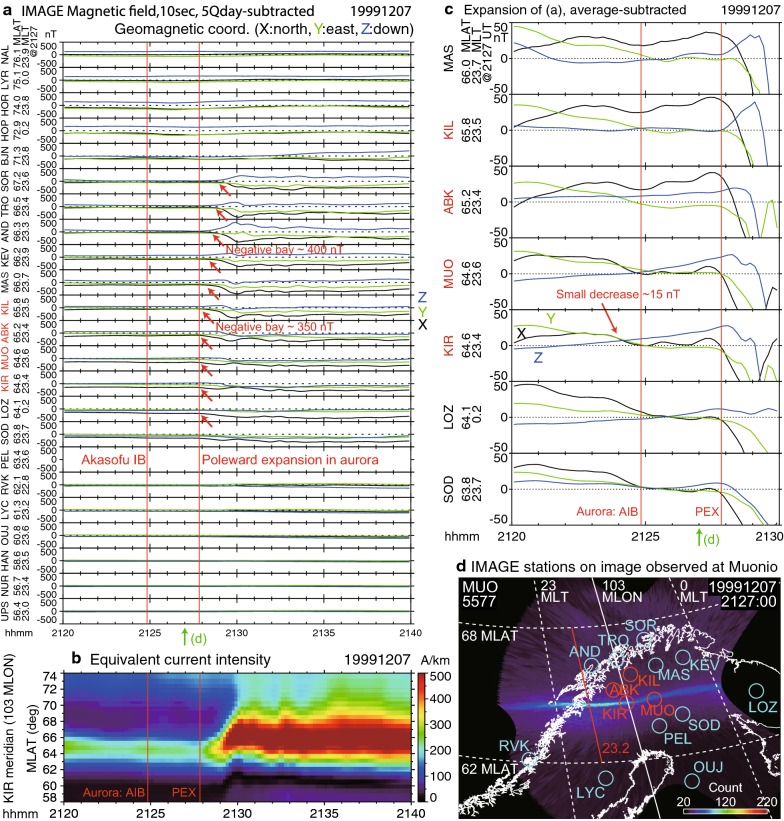
IMAGE ground magnetic observations near the substorm onset longitude in Europe. **a** Variations in the northward (*X*), eastward (*Y*), and downward (*Z*) components of the magnetic field in geomagnetic coordinates. The panels are presented in order of the observatory latitude, with the top panel corresponding to the highest magnetic latitude (MLAT) station. The magnetic local time (MLT) of each observatory at 2127 UT is shown at the left of each panel. Red vertical lines indicate the times of the Akasofu initial brightening (AIB) at 2124:50 UT and the poleward expansion at 2127:50 UT, both at [23.2 MLT, 64.6 MLAT] in the all-sky images (ASIs). The five-quiet-day baseline was subtracted for each observatory. The negative bay began at 2128 UT. **b** Intensity of the equivalent current at an altitude of 110 km, shown along the KIR station meridian ($103^\circ$ magnetic longitude). The equivalent current was derived by using the same data set as **a**, except that the van de Kamp (2013) baseline was used. **c** Expansion of **a** for weak variations beginning at 2124 UT. The average of the displayed interval was subtracted for each observatory. **d** Locations of some IMAGE magnetic stations overlaid on an ASI at Muonio captured during the AIB

Substorm onsets are also traditionally identified by using negative bays, positive bays, and Pi2 pulsations in ground magnetic field data. Figure 9 shows the negative bays with the 10-s resolution ground magnetic field data obtained from the International Monitor for Auroral Geomagnetic Effects (IMAGE) project (e.g., Viljanen et al. 1995; Tanskanen 2009). Figure 9a shows the northward (*X*), eastward (*Y*), and downward (*Z*) components of all available data in the geomagnetic coordinates. The Kiruna station (KIR: 64.6 MLAT, 102.7 MLON) was located at 23.4 MLT at the time of the AIB (2124:50 UT, the first red line). This location was close (Fig. 9d) to the AIB centered at [23.2 MLT, 64.6 MLAT]. However, no significant magnetic variations were detected at KIR and at other stations at the time of the AIB.

In contrast, the poleward expansion (Figs. 4 and 5) that began at 2127:50 UT (the second red line) was accompanied by decreases up to $\sim 400\hbox { nT}$ in the *X* component. The negative bays began around 2128 UT at the KIR and MUO stations near the onset MLAT, where the bays weakened temporarily after 2129 UT, presumably because the current center had moved poleward. The negative bay was more clearly observed just north ($65.2{^{\circ }}{-}65.8{^{\circ }}$: ABK and KIL) of the onset MLAT ($64.6^\circ$). Stations at higher latitudes ($66.3{^{\circ }}{-}66.5{^{\circ }}$: AND and TRO) detected sharp negative bays 1 min later at 2129 UT, and detected the largest decrease ($\sim 400\hbox { nT}$) among all stations at 2130 UT.

These magnetic field data were used to infer the equivalent electric current at an altitude of 110 km by using the method described in Juusola et al. (2016). We first derived the two-dimensional maps (not shown) of the equivalent current and then focused on the KIR station meridian at $103{^{\circ }}$ magnetic longitude, which was typically 0.2 h east of the auroral onset MLT center at 23.2 h. Figure 9b shows the time evolution of the inferred equivalent current intensity at this longitude. The equivalent current intensified around the time of the auroral poleward expansion, at 2127:50 UT. This intensification began around the auroral onset MLAT, at $64.6{^{\circ }}$, and then expanded poleward; these results are consistent with the latitudinal dependences of the observed magnetic variations shown in Fig. 9a.

The major negative bay of $\sim 400\hbox { nT}$ beginning at 2128 UT in Fig. 9a is considered to be a traditional substorm onset signature in the present study. However, it should be noted that much smaller variations are visible when the vertical scale is changed (Fig. 9c). Decreases in *X* began at 2124 UT near the onset latitude, $\sim 15\hbox { nT}$ at KIR and $\sim 20\hbox { nT}$ at MUO, corresponding to the weak enhancement in the equivalent current intensity at 2124 UT (Fig. 9b). These may be associated with the AIB (2124:50 UT about $\pm \,1\hbox { min}$), although it would be too weak to be identified conventionally as a substorm onset.

### Positive bays and Pi2 pulsations

**Fig. 10:**
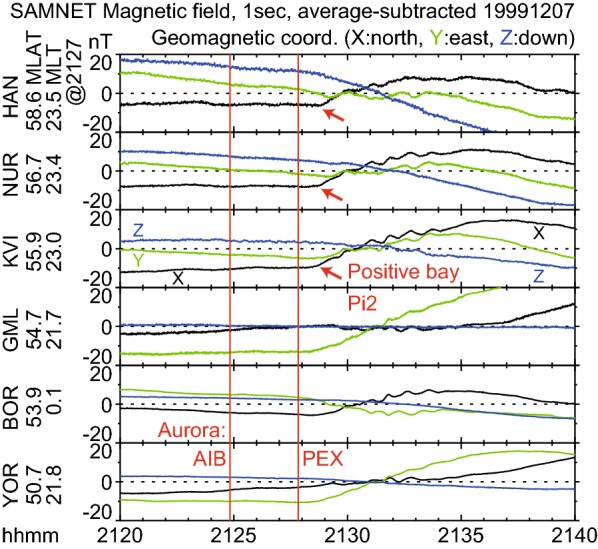
SAMNET ground magnetic observations near the substorm onset longitude below 60 MLAT. The format is the same as that of the IMAGE magnetic observations in Fig. 9a, except that the 1-s values are shown and the average of the displayed interval was subtracted for each observatory. Pi2 and positive bay began at 2128:50 UT

Figure 10 shows the 1-s resolution ground magnetic field data obtained through the Sub-Auroral Magnetometer Network (SAMNET) project (e.g., Yeoman et al. 1990), where stations below 60 MLAT were selected. Positive bays in the *X* component were evident at HAN, NUR, and KVI stations near the onset MLT, at 23.2 h. These positive bays started at about 2128:50 UT, which is $\sim 1\hbox { min}$ later than the poleward expansion but 4 min later than the Akasofu substorm onset.

Simultaneously, magnetic pulsations began at these stations. The peak-to-peak amplitude was approximately 3 nT with a periodicity of $\sim 50\hbox { s}$ inside the Pi2 range, at 40–150 s. Thus, the midlatitude Pi2 pulsations were observed in association with the poleward expansion. Associated Pi2 pulsations were observed at other stations (GML, BOR, and YOR), although their beginnings were less clear.

It should be noted that we concentrated on Pi2 pulsations at midlatitudes, where no aurora was observed in the GIs (Fig. 3a). Such Pi2s represent global magnetic variations and thus have been traditionally used as a substorm onset indicator. At auroral latitudes, Pi2-range variations may be observed at the time of the IB if the observatory is coincidentally located at the right place. However, the temporal and spatial variations of such auroral-latitude Pi2s are well correlated with local auroras (e.g., Rae et al. 2009). Thus, the implications of such auroral-latitude Pi2s differ from the lower-latitude Pi2s. Although the global variation component may be included in the Pi2-range variation at auroral latitudes, its extraction is difficult in the presence of auroras.

In summary, the geomagnetic signatures of the substorm onset were observed, including the start of development in AL, negative bay, positive bay, and midlatitude Pi2 pulsation. Such signatures began at about 2128–2129 UT, which is 0–1 min after the poleward expansion at 2127:50 UT but 3–4 min after the AIB at 2124:50 UT. Thus, these signatures do not likely correspond to the AIB; rather, they are more likely to be poleward expansion. The absence of significant geomagnetic responses to the AIB was also reported by Nishimura et al. (2012), Lyons et al. (2013), and Ieda et al. (2016). Lyons et al. (2013) further concluded that significant geomagnetic variations correspond to post-onset streamers from the poleward boundary of the auroral bulge. This detailed correspondence was difficult to confirm in this particular event, with the limited time resolution. Therefore, we conclude simply that significant geomagnetic variations correspond to poleward expansion.

## Discussion

In the present case study, two distinct auroral brightenings were observed in ground ASIs, as expected: the AIB and the following poleward expansion a few minutes later. This two-stage development is consistent with the classic Akasofu substorm onset (Akasofu 1964) and presumably corresponds to two different physical mechanisms.

**Fig. 11:**
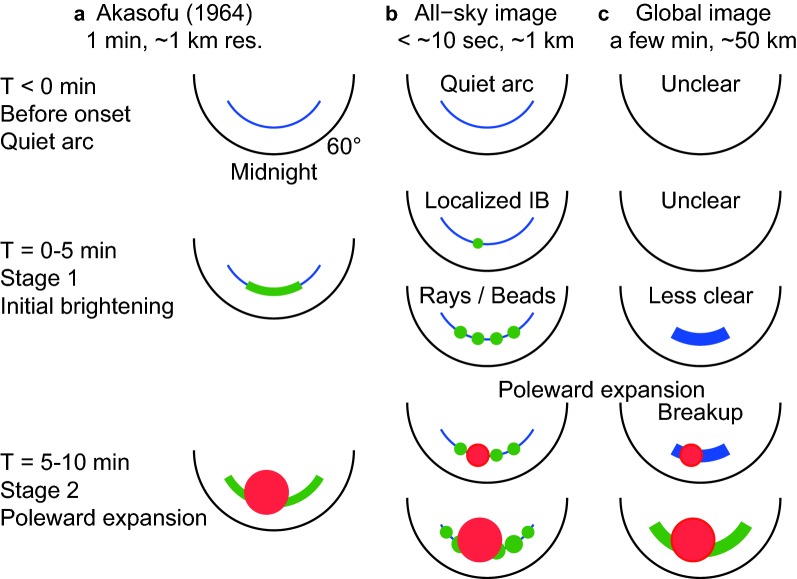
Synthesis of three different views of auroral substorm onset observations: **a** original concept (Akasofu 1964) based on 1-min resolution ground-based all-sky images (ASIs); **b** high time resolution ($< \sim 10~\hbox {s}$) ASIs; **c** satellite-based global images (resolution of a few minutes). The spatial resolution of ASIs ($\sim 1~\hbox {km}$) is much better than that of global images ($\sim 50~\hbox {km}$). From top to bottom, the time sequence of auroral emissions on the nightside ionosphere above $60^\circ$ magnetic latitude is illustrated. The blue, green, and red colors indicate weak, moderate, and intense recorded auroral emissions, respectively. The initial brightening (IB) is longitudinally extended in **a**. This IB may appear as localized at the beginning followed by rapid longitudinal expansion (auroral rays or auroral beads) in **b**, as indicated by green circles. Red circles indicate poleward expansion (i.e., auroral breakup). A substorm onset is identified by the IB in **a**, and practically by the poleward expansion in **c**. It is undecided whether the localized IB or the poleward expansion should be used to define the substorm onset in **b**. Auroral brightness is significantly underemphasized in global images, presumably by area averaging when the aurora is latitudinally thinner than the spatial resolution of the images

In contrast, the AIB, which was observed in the ASIs, was not evident in the GIs, as illustrated in Fig. 11. Consequently, the identified first brightening in the GIs corresponded to the second brightenings in the ASIs (i.e., the poleward expansion). In this section, we discuss these differences between ASIs and GIs, including time delay, causes, implications for the onset definitions, and impacts on the reconnection timing.

### Time delay of substorm onsets between ground and satellite images

In the present study, the substorm onset identified by using GIs was delayed from the ASI data by 3 min. This delay corresponds to the time difference between the AIB and the poleward expansion and thus corresponds to the duration of the first stage (Fig. 1b) of the substorm expansion phase in Akasofu (1964) of a few minutes. This Stage 1 often includes auroral rays (Akasofu 1964). We believe that auroral rays and auroral beads are different views of the same auroral structure and that both can be recognized as detailed features of a longitudinally wide brightening (i.e., AIB in Fig. 11).

The duration of the AIB in the ASIs was 2.5 min (Rae et al. 2009), a few minutes (Mende et al. 2009), and 7 min (Motoba et al. 2014) in previous case studies. The duration was 1–2 min on average and extended to 7 min in a statistical study (Nishimura et al. 2016). Thus, large diversity occurs in the identified delays/durations ($\sim 1{-}7\hbox { min}$). In the present discussion, we assumed that the time delay is typically a few minutes.

It is currently difficult to comprehensively understand this diversity, although a clue may be that the AIB tends to have a short duration when it intensifies rapidly (Nishimura et al. 2016). Practically, precursor brightenings are often observed prior to the AIB (e.g., Ieda et al. 2016). It is sometimes difficult to determine whether such a brightening is the AIB or a precursor, particularly when it does not decay significantly, leading to subjectivity in the duration of the AIB. Substorm onsets with a delay/duration shorter than the time resolutions of the GIs would appear to be simultaneous between the ASIs and GIs. Even in such cases, the implications of observed onsets are presumably different between the ASIs and GIs.

The delay of GI onsets from ASI onsets has been assumed to be small, at less than $\sim 1\hbox { min}$ (e.g., Liou 2010), without direct comparison of GIs and ASIs. Pi2s have been classical substorm onset signatures (e.g., Rostoker et al. 1980; Olson 1999; Nosé et al. 2012). GI onsets have been observed $\sim 1\hbox { min}$ prior to Pi2s (Liou et al. 2000). This correspondence may verify that the delays of GI onsets from ASI onsets are small. However, the present study and Ieda et al. (2016) suggest that major Pi2s are not likely associated with the Akasofu substorm onset, but rather with the poleward expansion later in the ASIs. Thus, the correspondence of GI onsets (i.e., poleward expansion) to Pi2s does not necessarily imply that the delays of GI onsets from ASI onsets are small. Rather, it suggests that the substorm onsets in the GIs are delayed with respect to the AIB in ASIs by more than that expected, depending on the duration of the AIB.

### Causes of differences between ground and satellite images

Poleward expansion was observed in both ASIs and GIs. This sudden change appeared to be even more evident in the GIs (Figs. 6b and 7b) than in the ASIs (Figs. 6a and 7a), indicating that the practical sensitivity of the GIs is sufficient to identify poleward expansion. In contrast, the AIB was not evident in the GIs, indicating that the sensitivity of GIs is considerably less than that of ASIs for identifying the AIB.

These results suggest that the different responses between ASIs and GIs may depend on the latitudinal thickness of the auroras. Our interpretation is that the brightness of the aurora is underemphasized when the target is thinner than the spatial resolution of images. This underemphasis is attributed to the averaging of an area that includes both the thin aurora and the adjacent dark region. The AIB is less evident in GIs, presumably because it is thin in terms of the latitude range, particularly at the beginning, compared with the spatial resolution of GIs. Thus, its brightness would be reduced significantly by area averaging. In contrast, the poleward expansion includes a thickening of the bright aurora; thus, its brightness would be reduced at the beginning but would not be reduced after the expansion has reached the spatial resolution of GIs. That is, the increase in brightness would be overemphasized in GIs when it begins to detect poleward expansion (i.e., auroral breakup).

Another possibility is that these different responses in ASIs and GIs may be attributed to the difference in wavelengths used to observe the auroras. The difference in wavelength did not result in significant differences in the brightness of the poleward expansion. However, it may contribute to difference in the brightness of the AIB. Both satellite (170 nm) and ground (557.7 nm) images are expected to be sensitive to precipitating electrons in the keV range. Thus, the difference in wavelength likely did not contribute significantly to the difference in observed auroras if the onset was dominated by keV-range electrons. However, precipitating electrons may belong to other energy ranges for the AIB. In such cases, the difference in wavelength may contribute to the different responses.

The AIB was not evident in the GIs in the present case; in other cases, the AIB may be sometimes visible in GIs depending on the conditions of auroras and cameras. However, the wide brightening is not explicitly included in identifications of substorm onset in GIs (e.g., Frey et al. 2004; Liou 2010), although it is not explicitly excluded. Thus, the AIB has not been typically recognized in GIs thus far. The AIB would be difficult to recognize as a substorm onset (i.e., sudden brightening) in GIs, not only because its brightness is underemphasized, but also because the increase in brightness of the following poleward expansion is overemphasized. With these assumptions, it may be sometimes possible to recognize a weak brightening in GIs as belonging to the AIB a few minutes prior to the major brightening (i.e., poleward expansion).

### Clarifications of substorm onset definitions

We have inferred that the traditionally identified onset brightening in satellite GIs does not necessarily correspond to the Akasofu substorm onset. Instead, it tends to represent the poleward expansion that follows a few minutes later (Fig. 11). Below, we discuss the reason why this interpretation has not been widely recognized.

#### Confusion regarding two different localized brightenings

Substorm onsets in GIs are traditionally identified by a localized brightening, which is labeled as auroral breakup (e.g., Frey et al. 2004; Liou 2010). Note that the two-stage development of the Akasofu model has not been required in these identifications, presumably because of the limited sensitivity of GIs. In contrast, this localized brightening in GIs is sometimes (e.g., Frank et al. 2001b; Morioka et al. 2014) specifically labeled as the (Akasofu) IB instead.

This confusion arises likely because it is not often recognized that the AIB (Akasofu 1964) is elongated along longitudes instead when considered on a timescale of a few minutes. This wide AIB may appear as localized ($\ll 1\hbox { MLT}\hbox { hour}$) at the very beginning ($\sim 10\hbox { s}$) in the ASIs (e.g., Liang et al. 2008) (Fig. 11). However, this weak aurora at the very beginning can be marginally recognized only on detailed inspection of ASIs; thus, it is expected to be barely detectable by GIs owing to the limited sensitivity and time resolution.

Moreover, such localized brightenings expand quickly in longitude, and the resultant wide aurora, sometimes including auroral beads, should be more evident than localized auroras. It is unlikely that the localized aurora at the very beginning was observed without observing the following brighter wide aurora. Thus, the observed localized brightening in GIs is unlikely to correspond to the localized brightening at the very beginning of the AIB in ASIs, at least in most cases.

As discussed above, the localized first brightenings in ASIs and GIs are not likely to represent the same phenomenon. This difference has not been often appreciated, likely also because both brightenings are referred to as “localized.” The first brightening in the GIs appears to be localized in wide-area images such as the 2128:07 UT panel of Fig. 3, but the same brightening does not appears to be localized in expanded images such as that in Figs. 6b and 5. Thus, the term “localized” has different implications between ASIs and GIs (Fig. 11) depending on the size of the displayed area.

#### Confusion regarding expansion onset and expansion phase onset

As discussed above, localized brightening in GIs is sometimes confused as corresponding to the Akasofu substorm onset. The same confusion arises likely because “expansion phase onset” sounds like the start of poleward expansion. One such example is a statement of (McPherron 2016): “The instant at which the aurora begins to expand poleward is called the onset of the expansion phase of the auroral substorm (Akasofu 1964).” This recognition is inconsistent with Akasofu (1964), as explained below.

A substorm is traditionally divided into three phases: the growth phase, the expansion phase, and the recovery phase. Substorm onsets refer to the beginning of the expansion phase (e.g., Baumjohann and Treumann 2012). The term “substorm onset” may be confused with the start of the growth phase and is often explicitly referred to as the “substorm expansion phase onset,” which is the beginning of the expansion phase, as this term itself defines.

The expansion phase is defined in Akasofu (1964) to begin with Stage 1 (AIB, i.e., without poleward expansion), followed by Stage 2 (poleward expansion) a few minutes later (Fig. 1). Thus, confusingly, there is no poleward expansion at the beginning of the expansion “phase” onset in the Akasofu substorm model. That is, “the instant at which the aurora begins to expand poleward” does not correspond to the expansion “phase” onset by definition.

#### Initial brightening or poleward expansion as a substorm onset

The two-stage development in the original definition of substorm onset has not been emphasized in later studies. For example, Rostoker et al. (1980) summarized various signatures to identify substorm onsets to include auroral arc brightenings, negative bays, positive bays, and Pi2s. Meng and Liou (2004) identified substorm onset as an auroral breakup, which they defined as a sudden brightening followed by poleward expansion. Such studies did not discuss these signatures in the context of the two-stage development; rather, they implicitly assumed only one stage.

In contrast, different stages have been used to define substorm onsets in recent studies. The AIB (i.e., the original definition, Stage 1) is sometimes adopted to identify substorm onsets (e.g., Donovan et al. 2008). Poleward expansion (i.e., Stage 2) is instead adopted with (e.g., Mende et al. 2009) or without (e.g., McPherron 2016) the recognition that this and the original definition differ. Substorm onsets in GIs are usually identified by the sudden brightening (e.g., Frey et al. 2004; Liou 2010). In contrast, Morioka et al. (2014) recognized in GIs that the sudden brightening is followed by another brightening a few minutes later; they identified the substorm expansion phase onset by this second brightening in GIs.

As summarized above, the definition of a substorm onset (i.e., substorm expansion phase onset) is currently diverging and is sometimes confused. To avoid such confusion, individual studies that include discussions within a few minutes of accuracy are recommended to state the definition of substorm onsets explicitly in the context of two-stage development. Two major possible definitions, AIB and poleward expansion, are discussed below.

If the substorm onset is defined as the first signature, it is likely to correspond to AIB, which is the original definition of onsets. Practically, this onset can be regularly monitored only by using ASIs. It may include auroral rays or beads and is often too evident to ignore before the beginning of poleward expansion. The AIB may be a manifestation of the triggering process of substorms, such as near-earth instabilities or the initial stage of tail reconnection. Even the AIB may play an active role in triggering substorms, for example, by feedback processes with the enhancement of ionospheric conductance and current. However, it may also be possible that the AIB is not directly associated with substorm onsets and occurs under background conditions favorable for the occurrence of substorm onsets.

In contrast, if the substorm onset is defined as the beginning of an explosive release of energy from the tail to the polar ionosphere, it is likely to correspond to poleward expansion. The poleward expansion presumably maps to dipolarization in the tail (e.g., Chu et al. 2015), thus manifesting the explosive energy release. Because the dipolarization is a drastic change in the magnetic field lines, it would cause major magnetic oscillations (i.e., major Pi2s). This onset can be identified by using various data sets such as GIs and geomagnetic fields in addition to ASIs and is thus useful at least as a working definition. However, it should be remembered that poleward expansion is not the original definition (Akasofu 1964) to time the substorm onsets.

### Impacts on past tail reconnection timing

Reconnection-associated fast plasma flows are often observed in the magnetotail near the time of a substorm onset identified by using Pi2s or GIs (Hones et al. 1984; Moldwin and Hughes 1993; Slavin et al. 2002; Ieda et al. 2008). These fast flows have occasionally been further identified a few minutes prior to the substorm onset (Nagai et al. 1998; Ohtani et al. 1999; Baker et al. 2002; Kepko et al. 2004; Miyashita et al. 2009).

However, such conclusions depend on the definition of substorm onset. Whether the identified substorm onset corresponds to the AIB or poleward expansion in ASIs has not been specified in these previous studies. In the present study, the onsets in Pi2s and GIs corresponded to poleward expansion rather than the Akasofu substorm onset. This result suggests that unless the longitudinally wide AIB was explicitly considered, the substorm onsets identified in past studies did not correspond to the Akasofu substorm onset but rather to poleward expansion.

Fast flows have always been initiated within a few minutes of the isolated auroral breakup in GIs (i.e., poleward expansion) if the satellite was located near the onset MLT (Ieda et al. 2008). However, unobserved AIB may have occurred prior to the auroral breakup (i.e., poleward expansion). Thus, these fast flows may be delayed from the possible AIB, as was reported in a case study by Ieda et al. (2016). In summary, no evidence exists for reconnection-associated fast flows prior to the Akasofu substorm onset. Therefore, the developed reconnection does not likely trigger the Akasofu substorm onset.

Reconnection-associated fast flows may be associated with auroral streamers. Some brightenings (e.g., 2126:17 UT panel) occurred near 73 MLAT near the onset MLT sector in Fig. 3a and b. Interestingly, an auroral streamer was formed at 72 MLAT near the onset MLT simultaneously with the breakup (2128:07 UT panel). This simultaneous occurrence may be a coincidence, or it may suggest that the auroral breakup (i.e., poleward expansion) and tail reconnection occur simultaneously.

## Summary

We have emphasized that the original definition of a substorm onset (Akasofu substorm onset) includes two-stage development: the AIB, which is wide in longitude, followed by poleward expansion a few minutes later. This two-stage development was originally proposed on the basis of ASIs. It has been unclear thus far how this two-stage development is observed in satellite GIs, in which the time resolution and sensitivity are limited.

In the present study, we directly compared optical substorm onset signatures observed in GIs and ASIs for an event that occurred on December 7, 1999. We used ultraviolet GIs captured by the Polar satellite during a fixed-filter mode at 170 nm, enabling a high time resolution of 37 s to resolve the possible two-stage development. The 20-s resolution green-line ASIs in Finland, at 557.7 nm, were used for comparison. Our results and discussions are summarized as follows.

1. A substorm onset was observed in the ASIs. These observations are consistent with the Akasofu substorm model, as expected, because the two-stage development was evident: A longitudinally extended brightening was followed by poleward expansion a few minutes later. In contrast, two-stage development was not evident in the GIs, even with the high time resolution of 37 s in the present case. Instead, the onset and poleward expansion occurred simultaneously in the GIs, as was the case in previous studies with a practical time resolution of a few minutes.
2. A comparison of ASIs and GIs revealed that poleward expansion occurred simultaneously, or within 1 min; however, the AIB in the ASIs did not have a counterpart in the GIs. Consequently, the substorm onset identified by using GIs was delayed by 3 min from the onset identified by using ASIs. This result suggests that the substorm onsets in GIs represent the beginning of poleward expansion rather than the AIB.
3. Major geomagnetic negative bays, positive bays, and midlatitude Pi2 pulsations were observed within 1 min after the poleward expansion but 3–4 min after the Akasofu substorm onset. Thus, the classic geomagnetic substorm signatures represent poleward expansion rather than the Akasofu substorm onset. This result suggests that the substorm onsets identified in GIs and geomagnetic data correspond to the same phenomenon (i.e., poleward expansion) but not to the Akasofu substorm onset.
4. We discussed that substorm onsets identified in past studies do not necessarily correspond to the Akasofu substorm onset but to subsequent poleward expansions, unless the AIB in ASIs was considered. The AIB is underemphasized and the poleward expansion is overemphasized in GIs because of the limited spatial resolution of GIs. Accordingly, poleward expansion tends to be identified as the substorm onset in GIs even when the AIB is moderately visible in ASIs.
5. Poleward expansion is useful as a working definition of substorm onset because the AIB is not regularly monitored and can be gradual. It should be noticed that this definition using poleward expansion (i.e., Stage 2) is not the original definition (i.e., Stage 1) of substorm onset.
6. We also discussed that the causality between tail reconnection and substorm onset depends on the definition of substorm onset. In past studies, reconnection-associated fast flows have been observed simultaneously or, in rare cases, prior to the substorm onset. However, because these onsets were identified by Pi2s or GIs, they were likely to correspond to subsequent poleward expansion rather than Akasofu substorm onsets. Thus, classical fast flows are associated with substorm onsets if the substorm onsets are defined by poleward expansion, but may not be directly associated with substorm onsets if the substorm onsets are defined by the AIB.

## Conclusion

At least two different instances have been considered for substorm onset in previous studies: the AIB (the original definition) and the poleward expansion (auroral breakup). It is necessary to clarify which instance is selected to time the substorm onset to understand the time history of substorms, including tail reconnection. For this purpose, we proposed a working model (Fig. 11) to synthesize the three different views of substorm onset: in the original Akasofu model, ASIs, and GIs. In the present study, “GIs” specifically refer to images with a practical spatial resolution of $\sim 50\hbox { km}$ or slightly worse, such as Polar/UVI or IMAGE/FUV images.

We suggest that substorm onset identified by GIs represents poleward expansion rather than the AIB. Although the AIB may be visible, its identification as a substorm onset would be less convincing in GIs. The two-stage development is not evident in GIs because their spatial resolution is limited. The practical significance of these inferences depends on the duration and intensity of the AIB, which is currently not well established.

## Abbreviations

AIB: Akasofu initial brightening
ASI: Ground-based all-sky image
CCD: Charged couple device
FUV: Far ultraviolet imager onboard the IMAGE satellite
GI: Satellite-based global image
IMAGE project: International Monitor for Auroral Geomagnetic Effects project
IMAGE satellite: Imager for Magnetopause-to-Aurora Global Exploration satellite
IB: Initial brightening
LBHL: Lyman–Birge–Hopfield long
LBHS: Lyman–Birge–Hopfield short
MLAT: Magnetic latitude in degrees
MLON: Magnetic longitude in degrees
MLT: Magnetic local time in hours
MUO: Muonio in Finland
OMNI: Operating Missions as Nodes on the Internet
SAMNET: Sub-Auroral Magnetometer Network project
UVI: Ultraviolet imager onboard the Polar satellite
